# Misconception of ‘malignant’ and ‘scissor-like compression’ of interarterial course in anomalous aortic origin of a coronary artery: a case series

**DOI:** 10.1093/ehjcr/ytae380

**Published:** 2024-07-30

**Authors:** Jonathan Schütze, Anselm W Stark, Marius R Bigler, Lorenz Räber, Christoph Gräni

**Affiliations:** Department of Cardiology, Inselspital, Bern University Hospital, University of Bern, Freiburgstrasse, CH - 3010 Bern, Switzerland; Department of Cardiology, Inselspital, Bern University Hospital, University of Bern, Freiburgstrasse, CH - 3010 Bern, Switzerland; Department of Cardiology, Inselspital, Bern University Hospital, University of Bern, Freiburgstrasse, CH - 3010 Bern, Switzerland; Department of Cardiology, Inselspital, Bern University Hospital, University of Bern, Freiburgstrasse, CH - 3010 Bern, Switzerland; Department of Cardiology, Inselspital, Bern University Hospital, University of Bern, Freiburgstrasse, CH - 3010 Bern, Switzerland

**Keywords:** Coronary anomaly, Anomalous aortic origin of a coronary artery, Scissor-like, Interarterial course, Sudden cardiac death, Mini case series

## Abstract

**Background:**

The notion that the ‘interarterial’ segment of anomalous aortic origin of a coronary artery (AAOCA) is ‘malignant’ and ‘scissor-like’ compressed between the aorta and pulmonary artery (PA) is debated, owing to the lower pressure in the pulmonary system compared with that in the coronary system. However, data supporting or refuting this belief under stress conditions are lacking.

**Case summary:**

Three cases of right AAOCA with interarterial/intramural courses (52, 66, and 51 years old) were assessed. Invasively measured fractional flow reserve (FFR) under dobutamine was 0.85, 0.82, and 0.81, respectively. Intravascular ultrasound illustrated lateral vessel compression of the intramural course with a decrease of minimal lumen area (MLA) (i.e. 5.71–3.47 mm^2^, 5.88–4.00 mm^2^, and 5.85–4.06 mm^2^) under stress conditions with heart rates of 130, 140, and 150 b.p.m., respectively. Pulmonary artery pressure (PAP) increased from rest {s/d (m) [systolic/diastolic (mean)] 22/11 (15), 15/2 (5), and 24/6 (14) mmHg} to stress [s/d (m) 47/24 (36), 30/3 (11), and 36/22 (24) mmHg] and remained below aortic peak pressure (blood pressure, BP) rest [s/d (m) 116/64 (91), 94/48 (71), 99/53 and (62) mmHg]; BP stress [s/d (m) 142/63 (80), 123/63 (88), and 86/46 (62) mmHg]; coronary pressure (CoP) rest [s/d (m) 100/59 (80), 80/45 (62), and 83/47 (63) mmHg]; and CoP stress [s/d (m) 95/60 (69),101/54 (72), and 70/32 (50) mmHg].

**Conclusion:**

This case series challenges the assumption that the interarterial segment of AAOCA is scissor-like compressed by both the aorta and PA. The decrease in MLA and FFR under stress is due to the aorta’s unidirectional lateral compression on the intramural segment. Additionally, the term ‘malignant’ should not be universally applied to all AAOCA cases with an interarterial course, as not all result in haemodynamic significance.

**Learning points**

- The traditional ‘scissor-like mechanism’ involving compression between the aorta and pulmonary artery is questioned, with evidence suggesting a unidirectional compression of the intramural segment by the aorta.
- Haemodynamic changes in anomalous aortic origin of a coronary artery (AAOCA) during exercise may be attributed more to the intramural course and other anatomical features than to compression between the great vessels.
- Not all cases of AAOCA with an interarterial course lead to haemodynamically relevant changes, emphasizing the importance of individualized assessment based on anatomical features.

## Introduction

**Figure 1:**
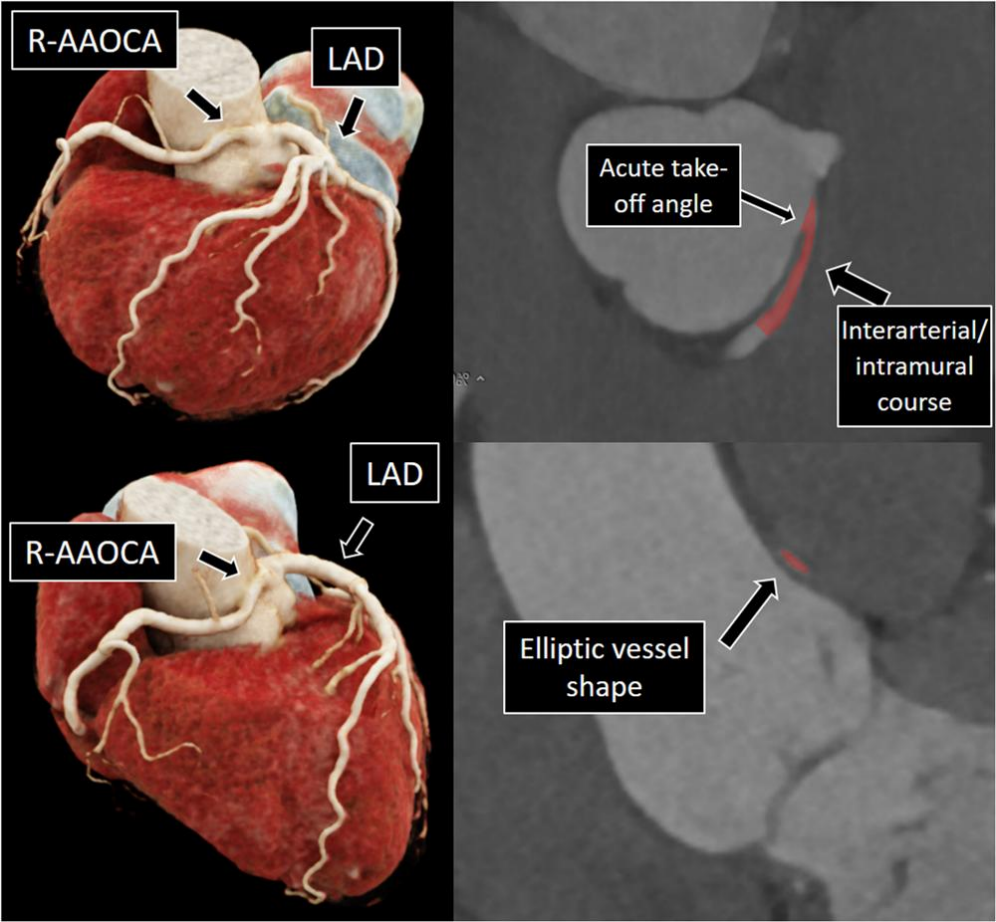
Right anomalous aortic origin of a coronary artery with the right coronary artery originating from the left coronary sinus demonstrating acute take-off angle and intramural course with elliptic vessel shape. AAOCA, anomalous aortic origin of a coronary artery; RCA, right coronary artery; LAD, left anterior descending artery.

Coronary anomalies are a rare congenital condition. A specific subgroup of coronary artery anomalies are anomalous aortic origin of a coronary artery (AAOCA) with an interarterial course, with a course of the proximal anomalous segment between the great arteries [aorta and pulmonary artery (PA)]^1,2^ and were found to be associated with ischaemia.^3–5^ Therefore, AAOCA with an interarterial course led to the historical classification of ‘malignant’ AAOCA, whereas those with a different course, namely, prepulmonic or retroarotic course, were called ‘benign’. As an explanation of ischaemia in AAOCA with an interarterial courses, a ‘scissor-like’ mechanism was suggested, which assumed the coronary vessel to be compressed between the aorta and the PA, especially under stress conditions.^3^ However, contemporary perspectives consider that the interarterial course rather acts as a surrogate marker for other anatomical high-risk features with pathophysiological consequences contributing to dynamic compression of the anomalous vessel segment during physical exertion.^6,7^ These include an acute take-off angle, lateral compression of the intramural segment (within the tunica media of the aorta), and a slit-like stenotic ostium (*Figure 1*). Among these, the slit-like ostium and the presence of an intramural course emerge as particularly concerning, especially when tachycardia and haemodynamic overload exacerbate myocardial demand while simultaneously reducing coronary flow^8–11^ (*Summary figure*). The historically presumed scissor-like mechanism implicated pressure exertion from both sides, the aorta and PA, creating a scissor-like effect on the anomalous coronary vessels segment in-between the great vessels.^12^ However, the logical coherence of this mechanism needs to be questioned, considering the typically lower pressure in the PA compared with the aorta and the coronary artery.^7^ Therefore, it is suggested that the mechanism causing lateral compression of the intramural course (within the aortic wall) is rather due to the unidirectional compression of the aortic wall itself. In this scenario, lateral compression is defined as the variation in the coronary artery’s lumen area in response to changes in aortic pressure, coronary pressure, and heart rate.^13^ The intramural segment, typically elliptic and narrower than a normal vessel, experiences heightened compression during systole and during exercise-induced pressure elevation.^5,14^ So far, no invasive measured data under different conditions are available that conclusively prove either mechanism. To address this uncertainty, we conducted a case series involving three patients with AAOCA with invasive assessment of pressures within the aorta (blood pressure, BP), PA (PA pressure, PAP), and anomalous coronary arteries during rest and stress conditions (coronary pressure, CoP), as well as fractional flow reserve (FFR, calculated as the ratio of the distal coronary pressure (Pd) to the aortic pressure (Pa) during hyperaemia: FFR=*Pd/Pa*). The exploration of these measurements under different conditions aims to improve the understanding of the intracoronary haemodynamics in AAOCA.

## Summary figure

**Figure:**
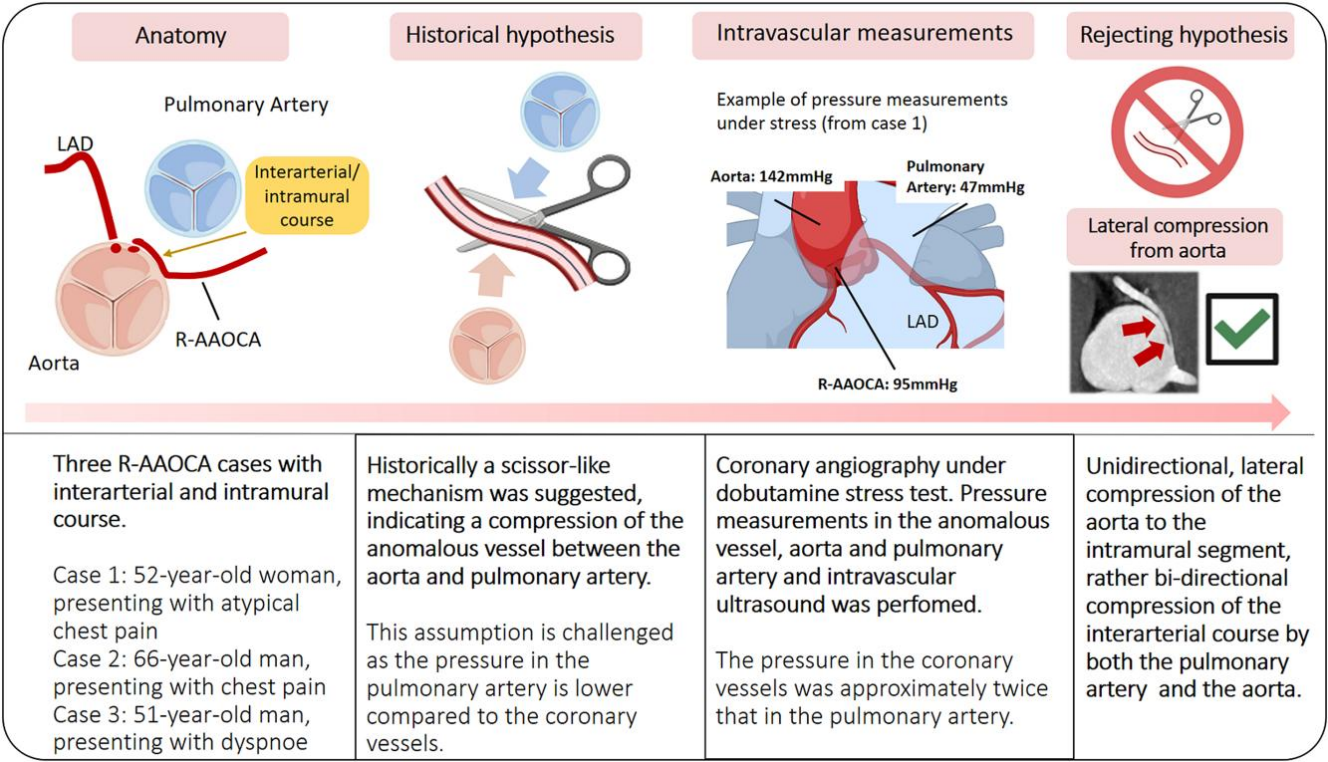
Graphical illustration of the evolvement of haemodynamic understanding in patients with AAOCA, demonstrating that the scissor-like compression of the anomalous vessel plays no role. AAOCA, anomalous aortic origin of a coronary artery; FFR, fractional flow reserve; IVUS, intravascular ultrasound images; LAD, left anterior descending artery; PA, pulmonary artery.

## Case presentation

We present three cases of Caucasian patients with right (R)-AAOCA and an interarterial and intramural course, all with right coronary dominance. Two were initially diagnosed using coronary computed tomography angiography (CCTA), whereas one case was diagnosed in invasive catheter (who underwent consecutive CCTA). All patients underwent testing for haemodynamic relevance of the anomalous coronary vessel using invasive coronary angiography with intravascular ultrasound (IVUS) imaging during rest and stress conditions with dobutamine–volume challenge (max. 40 μg/kg/min + saline-to-1.5–3 L + 1 mg atropine). Fractional flow reserve measurements were performed during dobutamine–volume challenge. The pulmonary pressure was additionally measured during rest and dobutamine–volume challenge as well as the aortic pressure and CoP (*Table 1*).

**Table 1:** Haemodynamic measurements in the three cases during rest and dobutamine stress in the pulmonary artery, aorta, and coronary vessel

Case	HR rest [b.p.m.]	HR stress [b.p.m.] (% of max. HR)	Maximum HR (220-age)	Delta HR rest/stress [b.p.m.]	BP rest [s/d (m) in mmHg]	BP stress [s/d (m) in mmHg]	Delta BP rest/stress [s/d (m) in mmHg]	PAP rest [s/d (m) in mmHg]	PAP stress [s/d (m) in mmHg]	Delta PAP rest/stress [s/d (m) in mmHg]	CoP rest [s/d (m) in mmHg]	CoP stress [s/d (m) in mmHg]	Delta CoP rest/stress [s/d (m) in mmHg]
1	67	150 (89)	168	83	116/64 (91)	142/63 (80)	26/-1 (−11)	22/11 (15)	47/24 (36)	25/13 (21)	100/59 (80)	95/60 (69)	−5/1 (−11)
2	81	130 (84)	154	49	94/48 (71)	123/63 (88)	29/15 (17)	15/2 (5)	30/3 (11)	15/1 (6)	80/45 (62)	101/54 (72)	21/9 (10)
3	70	140 (83)	169	70	99/53 (62)	86/46 (62)	−13/−7 (0)	24/6 (14)	36/22 (24)	12/16 (10)	83/47 (63)	70/32 (50)	−13/−15 (−13)

HR, heart rate; b.p.m., beats per minute; BP, blood pressure; s/d (m), systolic/diastolic (mean); PAP, pulmonary artery pressure; CoP, coronary pressure

## Case 1

**Figure 2:**
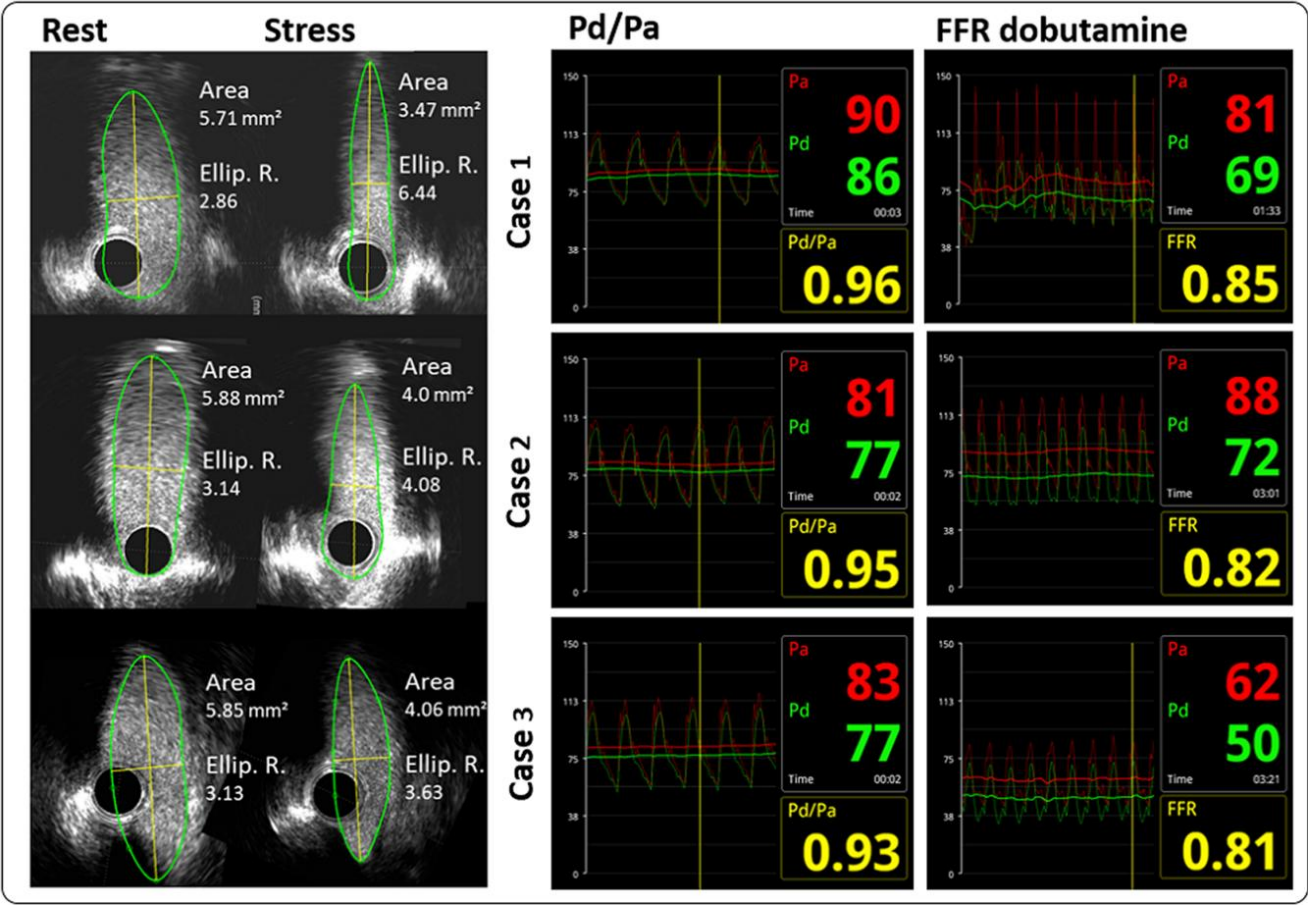
Intravascular ultrasound images of all three cases showing increasing ellipticity and decreasing lumen area under dobutamine stress (left) and fractional flow reserve curves of all three cases showing no sign of a relevant stenosis (right). FFR, fractional flow reserve; IVUS, intravascular ultrasound images; Pd/Pa, minimum distal coronary (Pd) to aortic pressure (Pa) ratio.

The first case was a 52-year-old woman, reporting long-standing thoracic discomfort. She described typical angina CCS II. Additionally, the patient experienced exertional dyspnoea classified as New York Heart Association (NYHA) functional class II–III. The patient suffered from various comorbidities including pulmonary arterial hypertension and obstructive sleep apnoea syndrome and had dyslipidaemia as cardiovascular risk factor. During the invasive evaluation for pulmonary hypertension, coronary artery disease (CAD) was ruled out and the patient was diagnosed with R-AAOCA and interarterial course. Coronary computed tomography angiography showed a slit-like ostium, and elliptical proximal vessel shape with an intramural length of 15 mm, a take-off angle of 12°, and proximal narrowing of 40% FFR dobutamine (heart rate 150 b.p.m., 89% of predicted max heart rate) demonstrated no relevant ischaemia, but a decrease of 0.96–0.85. The IVUS images indicated a lateral vessel compression of the intramural course with a decrease of the minimal lumen area (MLA) from 5.71 to 3.47 mm^2^ (delta 2.24 mm^2^) and a change of the elliptic vessel ratio from 2.86 to 6.44 (*Figure 2*). The aortic BP measurement at rest was normal {s/d (m) [systolic/diastolic (mean)] 116/64 (91) mmHg} and increased during stress [s/d (m) 142/63 (80) mmHg]. The PAP increased slightly [s/d (m) 22/11 (15) mmHg at rest to 47/24 (36) mmHg under stress] but remained well below the CoP, which in turn decreased from rest [s/d (m) 100/59 (80) mmHg] to stress [95/60 (69) mmHg].

## Case 2

The second case was a 66-year-old man, reporting recurring atypical chest pain over the past 20 years, which increased over the last year. Known cardiovascular risk factors were controlled arterial hypertension and controlled type 2 diabetes mellitus. Right AAOCA with an interarterial course was diagnosed in a CCTA with an elliptical proximal vessel shape with an intramural length of 20 mm, a take-off angle of 34.8°, a proximal narrowing with a 35.5% stenosis, but no slit-like ostium. Coronary artery disease was ruled out.

The FFR dobutamine measurements during mimicked exercise condition (heart rate 130 b.p.m., 84% of predicted max heart rate) did not reveal relevant ischaemia, but borderline decrease (0.95 to 0.82). The IVUS images indicated a lateral vessel compression of the intramural course with a decrease of the MLA from 5.88 to 4.00 mm^2^ (delta 1.88 mm^2^), while the elliptic vessel ratio changed from 3.14 to 4.08 (*Figure 2*). The aortic BP measurement at rest was [s/d (m)] 94/48 (71) mmHg and increased during stress [s/d (m) 123/63 (88) mmHg]. The pressure in the PA increased two-fold, [s/d (m) 15/2 (5) mmHg at rest to 30/3 (11) mmHg under stress] but also remained well under the CoP increasing under stress [s/d (m) 80/45 (62) mmHg at rest to 101/54 (72) mmHg under stress].

## Case 3

The third case was a 51-year-old man suffering from an increasing dyspnoea (currently classified as NYHA II) for the last 10 years, particularly noticeable during uphill activities. A CCTA ruled out CAD as cause for the symptoms, but a R-AAOCA with an interarterial course was diagnosed. The anomalous vessel showed a slit-like ostium and elliptical proximal vessel shape with intramural length of 11 mm, take-off angle of 28°, and a proximal narrowing of 33.3%.

The FFR dobutamine measurements during stress condition (heart rate 140 b.p.m., 83% of predicted max heart rate) did not reveal relevant ischaemia, but borderline decrease (0.93–0.81). The IVUS images did not show relevant changes of the MLA from 5.85 to 4.06 mm^2^ (delta 1.79 mm^2^) and a change of the elliptic vessel ratio from 3.13 to 3.63 (*Figure 2*). The aortic pressure measurement at rest was normal [s/d (m) 99/53 (62) mmHg] and decreased during stress [s/d (m) 86/46 (62) mmHg]. The pressure in the PA increased [s/d (m) 24/6 (14)  mmHg at rest to 36/22 (24) mmHg under stress], but just as in the first two cases, it remained well under the CoP decreasing under stress [s/d (m) 83/47 (63) mmHg at rest to 70/32 (50) mmHg under stress].

Given that (i) FFR under dobutamine stress did not reveal ischaemia (i.e. FFR > 0.8), (ii) dynamic MLA was not reduced by more than 50%, and (iii) fixed proximal narrowing degree (% area stenosis) was less than 50% on IVUS, the coronary artery anomaly was interpreted as an incidental finding without haemodynamic relevance in all three cases, and no further therapy was established. None of the patients experienced cardiovascular events during the 1-year follow-up period.

## Discussion

The case series provides insights about the complex interplay of anatomic features in AAOCA and its haemodynamic consequences. The historical classification of AAOCA with an interarterial course as being ‘malignant’, based on autopsy findings has shaped clinical perceptions within the last decades.^15^ It was perceived, that the interarterial course is bi-directional, scissor-like compressed from both sides (the aorta and the PA), especially during physical exercise.^7,12^ Firstly, contrary to the aforementioned misconception, our findings indicate that in patients with an interarterial course, the dynamic compression under stress is rather one-sided from the aortic side towards the intramural anomalous coronary segment, which is captured towards the other side by the outer aortic wall and the PA does therefore not play a role under stress conditions. This is supported by the fact that in the PA the pressure was always significantly lower compared with the aortic and the coronary artery pressure (even in the first case suffering from pulmonary hypertension). Notably, we observed only a moderate increase in pressure in the PA. However, since the pressure differences between the PA and coronary artery remained high, with the pressure in the coronary artery being twice that of the PA, the scissor-like mechanism is unlikely. Together with the decreased lumen area in the IVUS images, this further supports the hypothesis of aortic unidirectional lateral compression of the intramural segment as one of the leading contributing factors for haemodynamic changes within the anomalous vessel. Secondly, these case series showed that fixed proximal lumen narrowing and dynamic MLA changes did not lead to clinically significant ischaemia as assessed by FFR, and as such, the anomaly was classified as a non-relevant coincidental finding. Therefore, simply having an ‘interarterial’ course is not a proof of ischaemia under stress, reinforcing the idea that ‘malignant’ is an incorrect terminology for an interarterial course. In all patients with interarterial course, we suggest to instead assess the anatomical features individually, with the intramural course being one of the most critical factors. It is indeed possible for patients to present with an interarterial course without an intramural course. While an elliptical vessel shape is a mandatory feature for identifying the presence of an intramural course, the presence of an acute take-off angle (<45°) and slit-like ostium is frequent but not always observed in intramural courses. Additionally, it needs to be studied how interarterial compressions manifest in AAOCA patients with pulmonary hypertension and whether a scissoring-like mechanism can be observed in these rare specific cases.

As this is a case series, there are some obvious limitations such as the low patient number. Further, it should be noted that an oxygen wasting effect of dobutamine when used as stress agent was described, and ischaemia might occur at an earlier level than during true exercise.^16^ Unlike true exercise, dobutamine may not necessarily induce the same changes in vascular resistance, heart rate variability, and other physiological responses associated with physical exercise.^17–19^

## Conclusion

This case series challenges the historical belief that the interarterial segment of AAOCA is compressed in a scissor-like manner by the aorta and PA. The decrease in MLA and the decline in FFR of the anomalous vessel under stress are attributed to the unidirectional impact of the aorta to the intramural segment, rather to bi-directional compression of the interarterial course by both the PA and the aorta. Additionally, the term ‘malignant’ should not be universally applied to all AAOCA cases with an interarterial course, since not every case results in haemodynamic significance.

## Data Availability

Data is accessible upon reasonable request; however, the authors reserve the right to individually assess and decide upon each request.

## Lead author biography

Jonathan Schütze is a cardiologist who completed his medical training in Homburg, Germany, before pursuing further specialization at the University of Bern, Switzerland. His particular interest lies in cardiac imaging, where he is currently undergoing fellowship training.
